# Does the Order of Invasive Species Removal Matter? The Case of the Eagle and the Pig

**DOI:** 10.1371/journal.pone.0007005

**Published:** 2009-09-14

**Authors:** Paul W. Collins, Brian C. Latta, Gary W. Roemer

**Affiliations:** 1 Santa Barbara Museum of Natural History, Santa Barbara, California, United States of America; 2 The Bird Group, Santa Cruz, California, United States of America; 3 Department of Fish, Wildlife and Conservation Ecology, New Mexico State University, Las Cruces, New Mexico, United States of America; Stanford University, United States of America

## Abstract

**Background:**

Invasive species are recognized as a primary driver of native species endangerment and their removal is often a key component of a conservation strategy. Removing invasive species is not always a straightforward task, however, especially when they interact with other species in complex ways to negatively influence native species. Because unintended consequences may arise if all invasive species cannot be removed simultaneously, the order of their removal is of paramount importance to ecological restoration. In the mid-1990s, three subspecies of the island fox U*rocyon littoralis* were driven to near extinction on the northern California Channel Islands owing to heightened predation by golden eagles *Aquila chrysaetos*. Eagles were lured to the islands by an abundant supply of feral pigs *Sus scrofa* and through the process of apparent competition pigs indirectly facilitated the decline in foxes. As a consequence, both pigs and eagles had to be removed to recover the critically endangered fox. Complete removal of pigs was problematic: removing pigs first could force eagles to concentrate on the remaining foxes, increasing their probability of extinction. Removing eagles first was difficult: eagles are not easily captured and lethal removal was politically distasteful.

**Methodology/Principal Findings:**

Using prey remains collected from eagle nests both before and after the eradication of pigs, we show that one pair of eagles that eluded capture did indeed focus more on foxes. These results support the premise that if the threat of eagle predation had not been mitigated prior to pig removal, fox extinction would have been a more likely outcome.

**Conclusions/Significance:**

If complete eradication of all interacting invasive species is not possible, the order in which they are removed requires careful consideration. If overlooked, unexpected consequences may result that could impede restoration.

## Introduction

Invasive alien species are considered one of the most significant threats to biodiversity and on islands their impacts have been particularly grave [1]–[3]. As a consequence, the removal of invasive species has become an oft-used method to restore island ecosystems [4]. However, there are examples where removal of an invasive species has wrought unexpected and devastating results [5], [6]. Some examples of the unexpected consequences of removing an invasive species include the proliferation of exotic plants and increases in the abundance of subdominant, invasive predators that may then become a greater threat to the original targets of the restoration efforts [5]–[7]. Functional frameworks based on ecological principles (e.g., food web theory) can be used to presage unexpected consequences of removing invasive species, and pre-eradication data and/or ecological modeling coupled with continuous evaluation of program goals can further our knowledge of best practices for ecological restoration [6]. We were afforded a unique opportunity to assess how the order of removal of invasive species on the California Channel Islands could have influenced the recovery of the critically endangered island fox *Urocyon littoralis*.

Island foxes on Santa Cruz Island, California USA experienced precipitous declines in the mid-1990s owing to heightened predation by colonizing golden eagles *Aquila chrysaetos* [1], [8]. Although golden eagles were the proximate cause of the decline, feral pigs *Sus scrofa*, by acting as an abundant food lured golden eagles to the island and through the process of apparent competition indirectly caused the decline in foxes. Thus, removing both eagles and pigs were necessary management actions required to save the island fox. The question at the time was: Which one do you remove first?

A mechanistic model of this three-species interaction showed that if pigs were removed first, eagles could focus more on foxes possibly hastening their extinction [9]. Because it was increasingly difficult to capture the remaining eagles, lethal removal was advocated; a contentious suggestion that was never implemented [9]–[11]. Efforts to trap eagles were intensified, however, and new methods such as live capture with a net gun and helicopter were applied: between 1999 and 2006 a total of 44 golden eagles were removed [12], [13]. At the end of this effort (from 2005–2006) lethal removal of the feral pigs was completed [14]. Nevertheless, at least one pair of eagles – the Laguna Pair – eluded capture and nested on the island after pig removal. Here, using prey remains collected at golden eagle nests, we show that this pair intensified their take of island foxes after pig removal, validating the original model and showing that if eagle numbers had not been reduced first, fox extinction could have been a more likely outcome.

## Results

**Figure 1:**
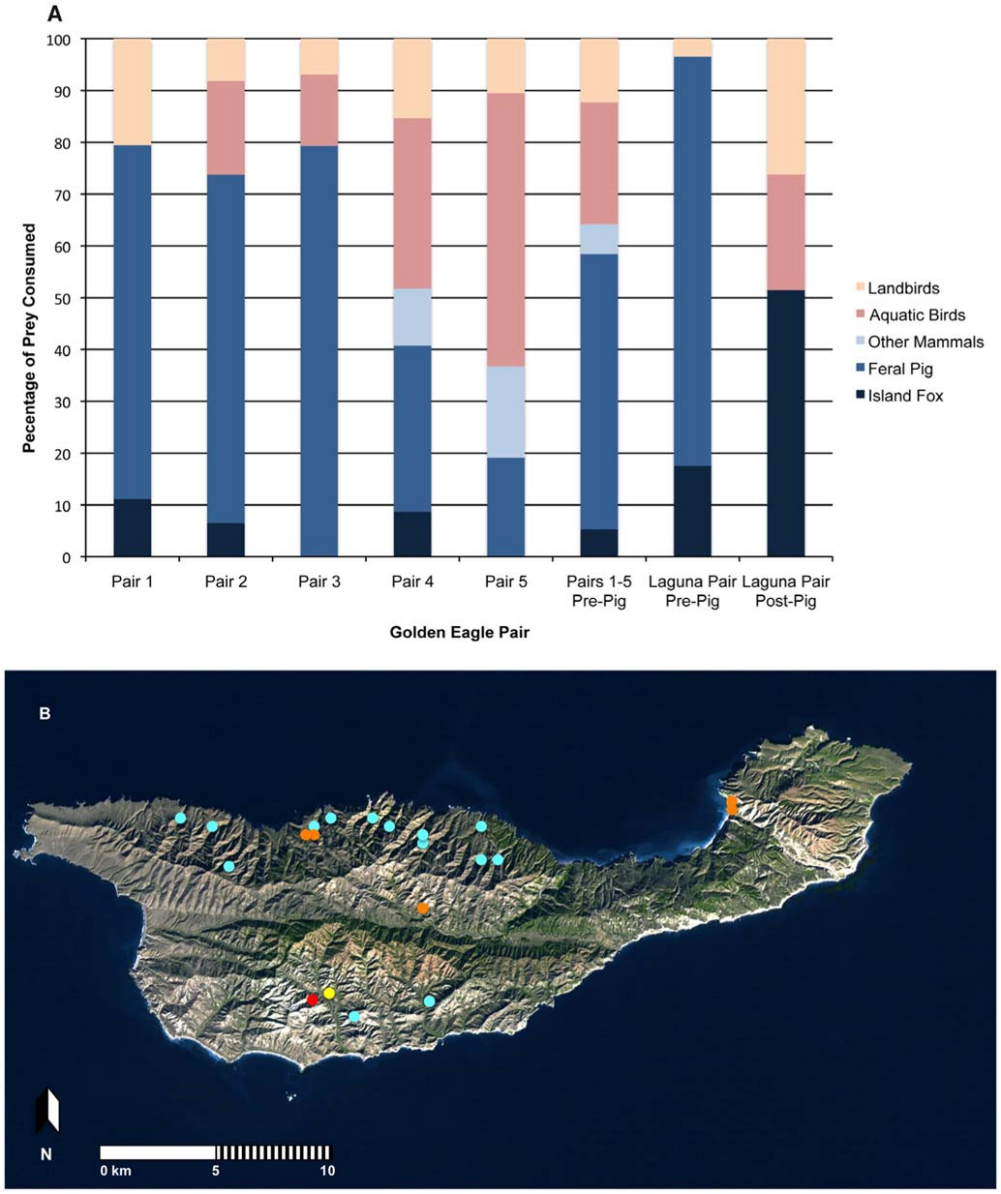
Prey remains and the distribution of golden eagle nests on Santa Cruz Island, California. (A) The percentage of prey biomass collected at five golden eagle nests excavated on Santa Cruz Island, California prior to eradication of the feral pig population and from the Laguna Pair pre- and post-pig removal. (B) The approximate locations of golden eagle nests on Santa Cruz Island. Shown are 14 suspected golden eagle nests (blue circles), the five different nests that were excavated (orange circles) and the two nests of the Laguna Pair, one prior to pig removal (yellow circle) and one after pig removal (red circle).

**Figure 2:**
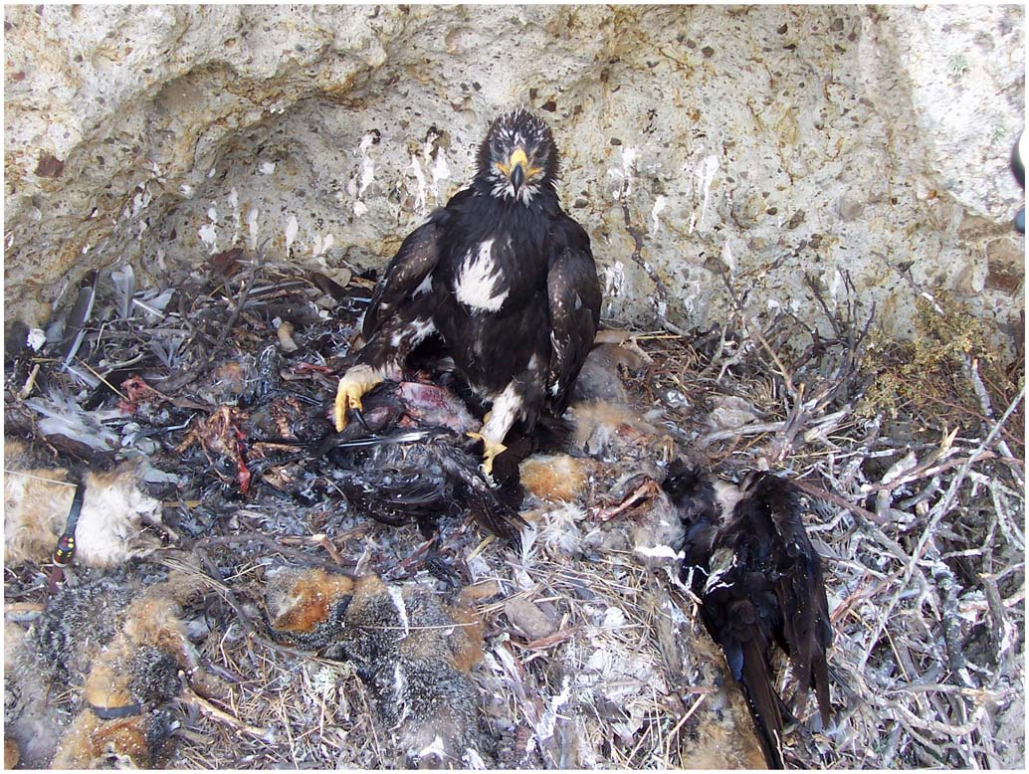
The nestling golden eagle of the Laguna Pair and her food. In June 2006, the nest of the Laguna Pair contained remains of 13 island foxes (note radio-telemetry collars), 11 common ravens and 12 seabirds (Photo credit: P. Sharpe).

The food habits of five nesting pairs of golden eagles prior to the removal of pigs but after the precipitous decline in foxes showed that piglets represented 41.1% of all individuals identified and 53.2% of total prey biomass, island foxes represented limited amounts, 5.1% and 5.3%, and birds comprised 51.3% of all individuals and 35.8% of the biomass, respectively (Figure 1A). Variation in food habits among the eagles was great; although all pairs consumed pigs, 4 of 6 pairs, including the Laguna Pair, consumed a lot of pigs (67.2 to 79.3% of biomass), others (Pairs 3 & 5) did not consume any foxes and still others (Pairs 4 & 5) consumed larger quantities of birds (48.1 and 63.2%, respectively). Collectively, the Laguna Pair had food habits that differed from the other nesting eagles (G = 16.7, d.f. = 4, P<0.005) primarily because they rarely preyed upon birds, consumed more foxes and relied more heavily on feral pigs. The Laguna Pair shifted their diet by dramatically increasing their consumption of foxes, from 17.5% to 51.5%, and native birds, from 3.4% to 48.5%, after pig removal (G = 63.0, d.f. = 4, P<0.001) (Figure 1A & Figure 2).

## Discussion

The original decline in foxes could have been caused by as few as seven eagles [8]. Because there were at least six nesting pairs on the island and most likely more (Figure 1B), had not the majority of eagles been removed island foxes could have gone extinct if pigs were removed first and if the remaining eagles focused more on native prey, including foxes, as the Laguna Pair had (Figure 2). Although our data are limited by having only a single nesting attempt available to examine after pigs had been removed, it nonetheless corroborates what would be the expected functional response of an apex, opportunistic predator like a golden eagle.

Golden eagles take a wide variety of vertebrate prey ranging in size from <65 g to >4 kg. Preferred prey often are highly fecund birds or mammals (e.g., rabbits or pigs) and when preferred prey are abundant golden eagles are specialists on them, when preferred prey are rare, they become generalist foragers and readily hunt alternative prey, including carnivores [15]. In Idaho, the composition of golden eagle diets was positively correlated with the abundance of black-tailed jackrabbits *Lepus californicus*, and when the density of jackrabbits declined, eagles switched to alternative prey [16]. On both Santa Cruz and Santa Rosa Islands, feral prey, in the form of either pigs or introduced deer and elk comprised substantial portions of the diet of colonizing golden eagles [17]. The observation of the Laguna Pair switching to native prey as pigs were eradicated is not only the predicted functional response for such a predator, but it also is the predicted functional response for an invasive predator when its invasive prey is removed first [6].

Although we believe that the shift in food habits toward native prey by the Laguna Pair was chiefly a consequence of the eradication of feral pigs, there are alternative potential explanations. For example: 1) the Laguna Pair may have always preferred foxes over pigs and with the concomitant removal of other eagles, which lowered eagle numbers and therefore increased fox numbers, there were more foxes available to the Laguna Pair; 2) the Laguna Pair was able to increase the proportion of foxes in their diet because eagle control removed adjacent pairs that excluded the Laguna Pair from prime fox hunting areas; and 3) as eagle numbers were reduced, foxes became less wary of eagles resulting in an increase in their vulnerability to eagle predation. We will attempt to address each of these alternative hypotheses in turn.

Fox numbers on Santa Cruz Island did increase from an estimated 137 individuals in 2001 to 264 foxes in 2006 [12]. These estimates represent very low fox densities (0.55 to 1.06 foxes/km^2^) and are 7x lower than fox densities just prior to eagle colonization of the island [18]. In contrast, 5,036 pigs (20.22 pigs/km^2^) were removed from the island in a period of ∼15 months [19]; although piglets are the eagle's prey, pigs were 20x more abundant than foxes and were removed in a relatively short period of time. The increase in foxes pales in comparison to the rapid eradication of pigs. Furthermore, the fact that eagle consumption of foxes was sustained at such low fox densities suggests a Type II functional response whereby predation related mortality would be highest for foxes when their densities are low; changes in pig density are expected to elicit a numerical response in the predator [18]. Thus, without pigs, eagles would have had to concentrate on whatever available prey there were, which was primarily foxes and native birds.

Eagle removal could have opened up prime fox hunting areas not previously accessible to the Laguna Pair. Prior to pig removal, three pairs of eagles (Pairs 1, 2 and 4) consumed quantities of foxes (6–11% of their diet) similar to, but still less than, the Laguna Pair (17.5%), suggesting that the Laguna Pair preferred foxes or already had prime fox hunting grounds. Prior to pig removal, the Laguna Pair occupied a territory on the south side of the island that was estimated to be ∼42 km^2^ [13]. We cannot truly evaluate this hypothesis as the size of Laguna Pair's territory after pig removal is unknown and we did not have access to data on the distribution of foxes during the relevant time period. However, a comparison of the size of golden eagle territories and movements during the breeding season may shed some light on the validity of this hypothesis. Eagle territories vary from as low as 1.5–9 km^2^ in the Balé Mountains of Ethiopia, to 49–152 km^2^ in San Diego County, California USA [20], [21]. These territory sizes are much smaller than Santa Cruz Island (249 km^2^) and in the latter study the largest ranges contained large portions of unusable agricultural land. In Scotland, average core area size was 48.1 km^2^ with most movements constrained to within 9 km of the home range center [22]. In Idaho, breeding season core areas varied from 0.3 to 1.5 km^2^ and individuals traveled an average of only 1.05 km (±0.37 km) from their nests during the nesting period [23]. Given the relatively small territory sizes of eagles compared to the size of the island, the even smaller core areas and the constrained movements typifying the nesting period, it seems unlikely that the Laguna Pair shifted its foraging area during the nesting period to any great degree especially because the nests used prior to and after pig eradication were located in the same canyon (Figure 1B).

Foxes may have become less wary and thus more vulnerable to the Laguna Pair. Previously, it had been suggested that foxes may have responded to the intense predation by reducing diurnal activity, either through experience (e.g., escaping a predation attempt) or because eagle predation acted as a selective force removing foxes that were more active during the day [1]. More recently, a comparison of activity patterns prior to and after the colonization of the island by eagles showed that wild foxes remaining on the island during the period of golden eagle occupation did reduce their diurnal activity [24]. The mechanism is still unknown, but these results suggest that wild foxes were less vulnerable to eagle predation by the time pigs had been removed; this was not true for captive-reared foxes, however. More importantly, our results suggest that even though foxes had reduced predation risk because of reduced diurnal activity, this reduction in risk was insufficient to offset predation related mortality after pig removal.

Currently, fox survival has increased above a critical threshold and fox populations are recovering, but eagles are still killing foxes even though the Laguna Pair has been captured [12], [25]. Because monitoring efforts to detect golden eagles have been reduced, it is possible that other golden eagles have gone undetected or still others have colonized the island from the mainland since pig removal [8]. Perhaps more intriguing is that the potential perpetrator of the recent mortalities of foxes is one or more bald eagles *Haliaeetus leucocephalus*. Recent necropsy evidence, expert opinion and the discovery of bald eagle feathers at fox carcasses on nearby Santa Rosa Island suggest that bald eagles may now be killing foxes [12], [25]. During the time that foxes were in decline, several management actions were implemented including the reintroduction of bald eagles [26]. It was hypothesized that bald eagles might act as a deterrent because bald eagles are highly territorial and may compete with golden eagles for nesting sites [8]; the efficacy of this management action was also hotly debated [10], [11]. Bald eagles had been extirpated from the Channel Islands by 1960 owing to a host of factors including the contamination of the surrounding waters with DDT [27]. Sixty-one bald eagles were released on Santa Cruz Island from 2002 to 2006 (P. Sharpe, pers. comm.) and at least two pairs have successfully fledged young there [26].

Although often thought of as being primarily piscivorous, mammals may make up to 14% of the diet of bald eagles [28]. On the Channel Islands, prey remains collected from historic bald eagle nests showed that they consumed both native and introduced mammals including island foxes [29]. Furthermore, video surveillance of reintroduced bald eagles on nearby Santa Catalina Island showed them bringing live feral piglets and goat kids to nests to feed dependent young (G. Roemer, pers. obs.); because the prey were alive, they had to have been captured by the parent eagles.

Our results corroborate earlier modeling efforts and reveal the value of modeling for forecasting extinction risk, especially when the nature of the interaction is known and reliable data are available for model parameterization [9]. Our results also point out how important natural history data can be to evaluating restoration efforts. By simply collecting and analyzing nest remains we've contributed to the validation of a predictive model and furthered our understanding of the mechanics of an eradication program. We do recognize, however, the lack of replication in our nest remains data, and thus encourage managers to seriously consider a rigorous approach to data collection when faced with such restoration activities. Finally, and perhaps most importantly, our results corroborate earlier important work that emphasized the need for management personnel to consider how native and invasive species interact, the order in which invasive species are to be removed and to anticipate the unanticipated [5], [6], [30]–[32]; untried management actions that at first seem very positive, such as the eradication of feral pigs or the reintroduction of bald eagles, may have hidden or unexpected consequences that require careful consideration.

## Materials and Methods

Between 2002 and 2006 prey remains were recovered from a total of seven golden eagle nests on Santa Cruz Island. The surface and areas immediately surrounding each nest were excavated by hand with the aid of trowels, shop brushes and a 1/16-in (1.59 mm) screen sieve. Prey remains recovered from different layers of the nest were combined. All excavations of nests were conducted with permission from both the state of California (Scientific collecting permit 801201-05) and the U.S. federal government (Federal bird banding permit 22383, USFWS permit MB017597-0).

Faunal remains were sorted into six taxonomic groups (fish, amphibians, reptiles, birds, mammals and invertebrates) and then identified to the highest taxonomic level possible by comparing diagnostic elements (e.g., bones, otoliths) to research specimens at the Santa Barbara Museum of Natural History. Fish, salamander and invertebrate remains were considered to be incidental remains that came into a nest either in the crops or stomachs of the prey of eagles, as riders on material used to line the nest cup, or by being attracted to decomposing remains in the nest; incidental faunal remains were excluded from diet analyses.

Two measures were used to calculate diet composition. First, the minimum number of individuals was determined for each species or taxonomic group to be equal to the greatest number of identical diagnostic elements per taxon. Second, a body weight value (biomass) was assigned to each species using published weight data. Because sex could not be reliably determined, we used the average weight of males and females of a given species. For feral piglets, we used an estimate of 2.5 kg, which represents an estimate of the maximum weight of a prey item that an eagle could be expected to transport back to its nest [15], [33].

Percent diet composition was calculated as the minimum number of all prey items in a given species or taxonomic group, divided by the total minimum number of all prey recovered, multiplied by 100. A similar method was used to calculate biomass using average body weights.

For analysis, we divided the prey items into five categories including the proportion of biomass of island fox, feral pig, other mammals (spotted skunk and feral sheep), land birds (principally common raven) and aquatic birds (cormorants and gulls). We then conducted two G-tests using the following observed and expected proportions of prey remains applied to the raw data: 1) comparison of the Laguna Pair to the average from the five other eagle nests prior to pig removal, and 2) comparison of the Laguna Pair post- and pre-pig removal.
